# BAG-1 predicts patient outcome and tamoxifen responsiveness in ER-positive invasive ductal carcinoma of the breast

**DOI:** 10.1038/sj.bjc.6604809

**Published:** 2008-12-09

**Authors:** E K A Millar, L R Anderson, C M McNeil, S A O'Toole, M Pinese, P Crea, A L Morey, A V Biankin, S M Henshall, E A Musgrove, R L Sutherland, A J Butt

**Affiliations:** 1 Cancer Research Program, Garvan Institute of Medical Research, Darlinghurst, Sydney, New South Wales 2010, Australia; 2 Department of Anatomical Pathology, South Eastern Area Laboratory Service, St George Hospital, Kogarah, New South Wales 2217, Australia; 3 Department of Medical Oncology, Westmead Hospital, University of Sydney, Westmead, New South Wales 2145, Australia; 4 Department of Pathology (SydPath), St Vincent's Hospital, Darlinghurst, Sydney, New South Wales 2010, Australia; 5 Department of Surgical Oncology, St Vincent's Hospital, Darlinghurst, Sydney, New South Wales 2010, Australia; 6 Faculty of Medicine, St Vincent's Clinical School, University of NSW, New South Wales 2052, Australia; 7 Division of Surgery, Bankstown Hospital, Bankstown, New South Wales 2200, Australia

**Keywords:** breast cancer, prognosis, response marker, BAG-1, tamoxifen sensitivity

## Abstract

BAG-1 (bcl-2-associated athanogene) enhances oestrogen receptor (ER) function and may influence outcome and response to endocrine therapy in breast cancer. We determined relationships between BAG-1 expression, molecular phenotype, response to tamoxifen therapy and outcome in a cohort of breast cancer patients and its influence on tamoxifen sensitivity in MCF-7 breast cancer cells *in vitro*. Publically available gene expression data sets were analysed to identify relationships between BAG-1 mRNA expression and patient outcome. BAG-1 protein expression was assessed using immunohistochemistry in 292 patients with invasive ductal carcinoma and correlated with clinicopathological variables, therapeutic response and disease outcome. BAG-1-overexpressing MCF-7 cells were treated with antioestrogens to assess its effects on cell proliferation. Gene expression data demonstrated a consistent association between high BAG-1 mRNA and improved survival. In ER+ cancer (*n*=189), a high nuclear BAG-1 expression independently predicted improved outcome for local recurrence (*P*=0.0464), distant metastases (*P*=0.0435), death from breast cancer (*P*=0.009, hazards ratio 0.29, 95% CI: 0.114–0.735) and improved outcome in tamoxifen-treated patients (*n*=107; *P*=0.0191). BAG-1 overexpression in MCF-7 cells augmented antioestrogen-induced growth arrest. A high BAG-1 expression predicts improved patient outcome in ER+ breast carcinoma. This may reflect both a better definition of the hormone-responsive phenotype and a concurrent increased sensitivity to tamoxifen.

Breast cancer is a heterogeneous disease with considerable variability in clinical outcome, the prognosis and management of which is largely based on histopathological features accompanied by established markers of hormone receptor status, oestrogen and progesterone receptors (oestrogen receptor (ER), progesterone receptor (PR)), and HER-2 amplification (Sorlie *et al*, 2001; Goldhirsch *et al*, 2007). Oestrogen receptor-positive disease comprises approximately 70% of cases and therapies targeting oestrogen synthesis or the ER are the most effective treatments, with adjuvant tamoxifen reducing the annual risk of recurrence and death by up to 47 and 26% respectively (Early Breast Cancer Trialists' Collaborative Group, 2005) and reducing the risk of contralateral disease by 50% (Fisher *et al*, 1998). However, the benefits of treatment are limited by intrinsic or acquired resistance, which occurs in approximately 40% of ER+ breast cancers (Howell *et al*, 2005). New predictive biomarkers of hormone responsiveness and disease outcome are needed to improve selection of patients for optimal ‘targeted’ endocrine therapy at an earlier stage in the disease process with potential survival benefits. In addition, they may also identify key mechanisms involved in antioestrogen resistance/sensitivity.

Gene expression profiling has identified intrinsic molecular phenotypes of breast cancer that subclassify ER+ tumours into two main subtypes that predict outcome: luminal A and B, which can be distinguished by the presence of increased proliferation, HER-2 amplification and a less favourable prognosis in the latter group (Sorlie *et al*, 2001). Signatures that predict outcome in ER+ disease treated with tamoxifen (Ma *et al*, 2004; Jansen *et al*, 2005; Loi *et al*, 2008) have been useful in identifying potential new predictive biomarkers. However, such molecular testing is expensive and there is often little overlap between signatures from different studies. Furthermore, translating these findings into clinically useful biomarkers suitable for routine pathology practice is a priority. Ideally, this would be performed using immunohistochemistry, which is more cost-effective and more easily introduced within the existing infrastructure. However, this approach is often limited by the lack of commercially available antibodies for many of these genes.

BAG-1 (bcl-2-associated athanogene) is a pro-survival protein that can influence diverse biological processes including nuclear hormone receptor function, apoptosis, signal transduction and protein turnover (reviewed in Cutress *et al* (2002)). BAG-1 exists as three protein isoforms. The specific ability of the long isoform, BAG-1L (p50), which possesses a nuclear localisation sequence not present in the other isoforms, to upregulate the transcriptional activity of both ER*α* and ER*β* up to five-fold in MCF-7 breast cancer cells (Cutress *et al*, 2003), is of potential functional and prognostic significance. BAG-1 is expressed in most normal human tissues (Takayama *et al*, 1998), and its overexpression has been described not only in breast cancer, but also in other human malignancies including squamous cell carcinoma of the head and neck (Shindoh *et al*, 2000), chronic lymphocytic leukaemia (Kitada *et al*, 1998) and prostate cancer (Maki *et al*, 2007), in which it is associated with a poor prognosis. However, its role as a predictive marker in breast cancer has not been established. Several studies have attempted to relate BAG-1 protein expression to disease outcome with inconsistent results, which may have been the result of low patient numbers, low rates of ER+ tumours (ER+ rates of 35–52% rather than a currently expected rate of ∼70%) and incomplete pathological, clinical and treatment information. However, the improved prognosis associated with a high *BAG-1* expression has earlier been demonstrated in three studies although with differences in subcellular localisation of BAG-1 expression that is, cytoplasmic (Turner *et al*, 2001), nuclear (Cutress *et al*, 2003), and cytoplasmic or nuclear (Nadler *et al*, 2008). More recently, BAG-1 featured as one of the 16 cancer-specific genes included in the Oncotype Dx assay (Paik *et al*, 2004), which predicts distant failure in ER+, lymph node-negative patients treated with tamoxifen using PCR of formalin-fixed paraffin-embedded (FFPE) material. In addition, this assay has also been used to predict the potential benefit of chemotherapy (Paik *et al*, 2006) in this group of patients.

As ER-mediated regulation of cell growth, proliferation and survival are key components of breast cancer development, the role of BAG-1 as a predictive and prognostic marker in breast cancer requires further investigation. Consequently, we aimed to define the relationship of BAG-1 expression with outcome and response to therapy in a large cohort of early breast cancer patients of uniform histological type with well-documented treatment and follow-up data.

## Materials and methods

### BAG-1 mRNA expression and outcome

Publically available gene expression data from two published studies (van de Vijver *et al*, 2002; Naderi *et al*, 2007) of breast cancer outcome were analysed to initially identify a potential relationship between BAG-1 mRNA levels and prognosis. The cohorts chosen for these analyses were of similar clinicopathological composition to our clinical cohort. The study by Naderi *et al* (2007) comprised 135 patients, 70% of whom were ER+ with a median follow-up of 132 months (range 16–160 months). Data were generated using Agilent Human 1A arrays, which were available as raw scanner data files and sourced from Array Express (http://www.ebi.ac.uk/) accession E-UCON-1. Using the limma R package (R Development Core Team, 2007), background-subtracted data were normalised by the global LOESS technique applied to non-control spots only. To combine information from duplicate dye-swap arrays, a linear model was fitted to the normalised data using limma (Smyth, 2005). Model fit coefficients for each sample were then used as final expression estimates, expressed relative to a pooled reference RNA. The second data set, sourced from van de Vijver *et al* (2002), comprised 295 patients, 76% of which were ER+, with a median follow-up of 93.6 months (range 0.6–220 months). Data were generated using Rosetta NKI-spotted oligonucleotide arrays and were downloaded from http://microarray-pubs.stanford.edu/wound_NKI/explore.html as log 2-transformed values in a text table format. Raw data were directly transferred to the final output file without further processing. One BAG-1 probe set was available from each cohort and expression data were analysed for frequency distribution of mRNA and its association with patient outcome.

### Patient characteristics

BAG-1 protein expression was assessed by immunohistochemistry in tumours from a cohort of 292 patients diagnosed with invasive ductal breast carcinoma and treated by a single surgeon (PC) between February 1992 and August 2002. Formalin-fixed, paraffin-embedded tissue was retrieved from St Vincent's Public Hospital (Sydpath) and St Vincent's Private Hospital (Douglas Hanly Moir Pathology), Sydney, Australia. All tumours were classified as invasive ductal carcinoma of no special type and graded using standardised histological criteria (Elston and Ellis, 1991). Lymph node status was assessed by axillary sampling and histological examination. Follow-up intervals were calculated from the date of definitive procedure (biopsy/lumpectomy/mastectomy) to the date of last-recorded follow-up (median 64 months, range 0–152 months). Patients less than 50 years of age with node-positive, ER− tumours or tumours larger than 3 cm received adjuvant chemotherapy (cyclophosphamide, methotrexate and 5-fluorouracil or adriamycin and cyclophosphamide (AC)). Patients with ER+ tumours who were more than 50 years of age received 5 years of tamoxifen therapy. Breast cancer-specific survival was defined as date of definitive procedure to date of death due to breast cancer. Patients who died of causes unrelated to breast cancer were considered as censored at the time of death. Deaths from unknown causes were excluded from analysis of disease-specific survival. Recurrences were confirmed by imaging and/or histology. Locoregional recurrences were defined as of the ipsilateral breast, chest wall, axilla or supraclavicular fossa. Distant relapses and metastases were defined as disease in the lungs, liver, brain or distant lymph nodes. These data were obtained from annual review of patient files or cancer registry data. Tissue microarrays (TMAs) of FFPE tumour tissue blocks were constructed with approximately 80 × 1 mm cores per slide. Each patient was represented by two to six 1 mm cores. Prior approval for this study was obtained from the Human Research Ethics Committee of St Vincent's Hospital, Sydney (HREC SVH H94/080, HREC 06336 SVH H00 036).

### Immunohistochemistry

Four-micron sections were cut from each TMA, mounted on SuperFrost® Plus glass slides and baked for 2 h at 79°C, then dewaxed by passage through xylene (two 5 min washes), cleared and rehydrated in graded alcohol (100, 95 and 70%) ending in a distilled water wash. Antigen retrieval was performed using DAKO solution (pH 6.0) (s1699; DAKO, Carpentaria, CA, USA) in a pressure cooker (DAKO Pascal Decloaker) for 60 s, followed by cooling gently for 15 min in a running water bath. Following a thorough wash in distilled water, endogenous peroxidase activity was eliminated with 3% hydrogen peroxide for 5 min. Slides were incubated with BAG-1 mouse monoclonal antibody raised against full-length human BAG-1 protein (clone 3.10G3E2; Santa Cruz Biotechnology Inc., Santa Cruz, CA, USA) at a dilution of 1 : 50 for 45 min at room temperature. Following buffer wash, detection employed DAKO Envision+ mouse secondary reagent (DAKO) for 30 min at room temperature, followed by DAKO DAB+ chromagen (DAKO) for 10 min. Slides were then rinsed in water and counterstained with haematoxylin, dehydrated through graded ethanol, cleared in xylene and mounted. Normal colon was employed as a control tissue that showed positive staining in basal crypt cell nuclei and negative staining in the muscularis mucosae. A further negative control substituted isotype-matched mouse IgG1 at 1 : 100 in place of the BAG-1 monoclonal antibody. Oestrogen receptor, PR, cytokeratin 5/6 and EGFR were also stained using the following antibodies: ER, 1 : 100 (clone 6F11; DAKO); PR, 1 : 200 (clone PgR 636; DAKO); CK5/6, 1 : 80 (clone MAB1602; Chemicon International, Temecula, CA, USA); and EGFR, 1 : 100 (clone H11; DAKO). HER-2 FISH was assessed in the Australian National Reference Laboratory (Department of Pathology, St Vincent's Hospital, Sydney) using the Vysis PathVysion HER-2 DNA dual-colour probe kit. An HER2 : chromosome 17 ratio>2.2 was classified as HER2 amplification.

All assessments of immunohistochemical staining were performed by observers blinded to the clinical and molecular data and patient outcome. Nuclear and cytoplasmic staining for BAG-1 was assessed by an experienced breast pathologist (EKAM) and described in terms of the intensity (0: negative, 1+: weak, 2+: moderate and 3+: strong) and percentage of cells staining positive. From these indices, a simplified ‘*H* score’ (i.e., intensity × percentage of positive nuclei) was calculated for each core and a mean and median score for each parameter calculated for each tumour (range of two to six cores per patient). Oestrogen receptor and PR (both double scored) were assessed as positive if they had a simplified *H* score of >10. CK5/6 and EGFR (both double scored) were assessed as positive if there was any positive cytoplasmic or membranous staining present at any intensity.

### Definition of intrinsic molecular phenotype of breast cancer

This was assessed immunohistochemically using criteria similar to those recently described by Cheang *et al* (2008) but using FISH to determine HER-2 status as follows: luminal A=ER+ and/or PR+, HER-2−; luminal B=ER+ and/or PR+, HER-2+; HER-2=ER− and PR−, HER-2+; basal-like=ER−, PR−, HER-2−, CK5/6+ and/or EGFR+; unclassified=negative for all five markers.

### Cell culture studies

The human ER+ breast cancer cell line, MCF-7, was routinely maintained in RPMI-1640 medium supplemented with 5% foetal calf serum, 10 *μ*g ml^−1^ insulin and 2.92 mg ml^−1^ glutamine under standard conditions. A cDNA insert encoding human BAG-1 (cat no. SC107955; OriGene Technologies Inc., Rockville, MD, USA) was cloned into the retroviral vector pMSCV-IRES-GFP (Caldon *et al*, 2008). MCF-7 cells transiently expressing the murine ecotropic receptor were infected with BAG-1 retrovirus as described earlier (Debnath *et al*, 2003). Green fluorescent protein-positive cells were sorted to homogeneity by flow cytometry. Cell lysates were collected as described earlier (Prall *et al*, 1997). Subsequent western blotting using a BAG-1 antibody (3.10G3E2; Clone Chemicon International Inc., Billerica, MA, USA) confirmed BAG-1 expression. *β*-Actin (Sigma, St Louis, MO, USA) was used as a loading control.

### S-phase analysis

Exponentially growing MCF-7 cells expressing BAG-1 or vector-alone control were treated with 1 *μ*mol l^−1^ 4-hydroxytamoxifen (Sigma) or 10 nmol l^−1^ ICI 182780 (7*α*-[9-(4,4,5,5,5-pentafluoropentylsulphinyl) nonyl] estra-1,3,5,(10)-triene-3,17*β*-diol), which was a kind gift from Dr Alan Wakeling (Astra-Zeneca Pharmaceuticals, Alderly Park, Cheshire, UK), or vehicle (ethanol) for 24 h. Cells were harvested and S phase was analysed by propidium iodide staining and flow cytometry.

### Statistical analyses

Statistical analyses were performed using Statview 5.0. Software (Abacus Systems, Berkeley, CA, USA). A *P*-value <0.05 was accepted as statistically significant. BAG-1 mRNA and protein expression and its association with clinicopathological variables and intrinsic molecular phenotype of breast cancer were tested by applying the *χ*^2^-test of association in contingency tables. Kaplan–Meier and Cox proportional hazards model were used for univariate analysis and the latter for multivariate analyses. Those factors that were prognostic in univariate analysis were then assessed in a multivariable model to identify factors that were independently prognostic and those that were the result of confounding variables.

## Results

### BAG-1 mRNA expression and outcome

**Figure 1:**
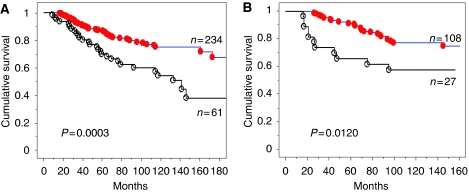
Relationship between BAG-1 mRNA expression and patient outcome. Kaplan–Meier analysis (log-rank test) for breast cancer-specific death in the Wound/NKI (**A**) and Naderi (**B**) cohorts. High BAG-1 (•); low BAG-1 (○).

To identify an association between BAG-1 gene expression levels and patient outcome, we examined two published breast cancer gene expression data sets. A frequency distribution of BAG-1 mRNA expression was used to apply serial cut points using sequential Kaplan–Meier analysis (log-rank test) to minimise the *P*-value and maximise the difference in survival between the two groups of high and low expressions. Using this approach, statistical significance was assessed for death using univariate Kaplan–Meier and Cox proportional hazards analysis (Table 1). The Wound/NKI data set of 295 patients contained a high BAG-1 expression group of 234 patients (79.3%), which was associated with improved prognosis in Cox and Kaplan–Meier univariate analysis (*P*=0.0005, Table 1A and Figure 1A). High BAG-1 expression was not significant in a multivariate model that incorporated standard clinicopathological variables (Table 1B). The Naderi cohort of 135 patients contained a high expression group of 108 patients (80%), again associated with a favourable outcome in Kaplan–Meier (*P*=0.0120) and Cox univariate analyses (*P*=0.0151, Table 1A and Figure 1B) but not in multivariate analysis (data not shown). Using these cut points to define high and low expression, further analyses were conducted to determine association between high BAG-1 expression and clinicopathological parameters. In the Wound cohort, high BAG-1 was associated with ER+, PR+, low histological grade and HER-2 negativity (*P*<0.0001) but there was no association with tumour size (*P*=0.0862) or lymph node status (*P*>0.999). Similarly, the Naderi cohort also showed positive associations between high BAG-1 expression and ER+ (*P*=0.0014), HER-2 negativity (*P*=0.0044) and low histological grade (*P*=0.0061) but not with tumour size or lymph node status (*P*=0.081 and *P*=0.106, respectively). These findings support an association of high BAG-1 expression with a luminal A phenotype and improved survival.

### Immunohistochemical analysis of BAG-1 protein expression in normal breast tissue and invasive ductal carcinoma

**Figure 2:**
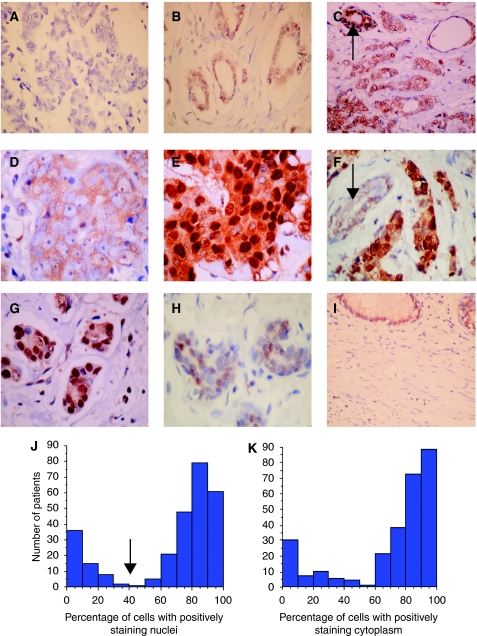
Representative images of BAG-1 immunohistochemistry. (**A**) Negative staining in high-grade invasive ductal carcinoma (IDC), × 400. (**B**) Weak (1+) nuclear staining in low-grade IDC, × 400. (**C**) Moderate (2+) nuclear and weak (1+) cytoplasmic staining in low-grade IDC, with strong nuclear staining in an adjacent normal duct (arrow). (**D**) Moderate (2+) cytoplasmic and negative nuclear staining in high-grade IDC. (**E**) Strong (3+) nuclear and moderate (2+) cytoplasmic staining in high-grade IDC. (**F**) Strong (3+) nuclear and weak (1+) cytoplasmic staining, weak nuclear staining in normal duct (arrow), × 400. (**G**) Strong 3+ nuclear staining. (**H**) Moderate nuclear staining in normal acini. (**I**) Normal colon, control tissue, which shows moderate positive nuclear staining in basal crypt cell nuclei and negative staining in mucularis mucosae. Frequency distribution of BAG-1 nuclear (**J**) and cytoplasmic (**K**) staining using immunohistochemistry in 276 invasive ductal carcinomas. There are two distinct populations that can be dichotomised using a cut point of 40% (arrow), which segregates the cohort into high- and low-expressing subgroups.

Representative immunohistochemistry staining patterns and intensities of BAG-1 are illustrated in Figure 2A–I. Similar patterns of staining were observed in normal terminal duct lobular units adjacent to cancer (*n*=24, 20 patients) and in reduction mammoplasty specimens (*n*=20, 14 patients). Nuclear staining was observed in all cases, with a mean of 54% of epithelial cells (range 10–90%) showing weak-to-strong (1–3+) intensity. Cytoplasmic staining was also present in 63% of cases with a range of 0–100% of cells showing 1 or 2+ staining.

In our cohort of 292 patients, 276 invasive ductal carcinomas were available for BAG-1 analysis due to loss of some tissue cores during processing of the TMAs. Staining was of variable intensity, which ranged from negative to strong (0–3+) and demonstrated both cytoplasmic and nuclear staining in keeping with the known subcellular localisation of the various BAG-1 isoforms (Cutress *et al*, 2002). There was, however, no direct correlation between nuclear and cytoplasmic expression when modelled as continuous variables (*R*=0.476). Sixteen out of 276 cases (5.7%) showed no nuclear staining, whereas 26 cases (9.4%) showed no cytoplasmic staining. When assessed for the percentage of positively staining nuclei, there appeared to be two distinct sub-populations, which could be dichotomised at a cutoff value of 40% positively staining nuclei at any intensity (Figure 2J). Cytoplasmic staining displayed a similar pattern (Figure 2K), with most tumours showing at least weak positivity but again with two distinct populations that could be identified using a 40% cutoff value. By applying the selected cut point of >40% mean nuclear staining, we defined 78% (214 out of 276) of the cohort as ‘high’ BAG-1 expressers and 22% (62 out of 276) as ‘low’ BAG-1 expressers. This cut point appeared to represent a real split in the protein expression data, which matched that observed from our analysis of the mRNA expression levels. This distribution was not apparent in the frequency distribution of nuclear ‘*H*’ scores. Consequently, we adopted the percentage of positively staining nuclei as the index for further analysis of association with outcomes.

### Correlation of BAG-1 expression with clinicopathological features and intrinsic molecular subtype

The relationship between nuclear and cytoplasmic expression of BAG-1 and standard clinicopathological features of the disease are summarised in Table 2A. High expression of BAG-1 showed a significant positive correlation with low histological grade, ER and PR positivity (*P*<0.0001) and was correlated negatively with HER-2 amplification status (*P*=0.001) and the triple-negative phenotype (*P*<0.0001). These findings were apparent for both nuclear and cytoplasmic staining at a cut point of 40% positivity of any intensity, but with a higher degree of statistical significance for nuclear staining. High nuclear BAG-1 expression was also strongly correlated with a luminal A intrinsic phenotype: 73% (154 out of 211) of BAG-1 ‘high’ were luminal A (*P*<0.0001, *χ*^2^-test), but there was no correlation with luminal B (*P*=0.956). A strong negative correlation was observed with the HER-2 (5%, 11 out of 213 BAG-1 high are of HER-2 phenotype, *P*<0.0001) and the basal-like phenotype (7%, 14 out of 213 BAG-1 high are basal-like, *P*<0.0001).

### BAG-1 expression and outcome

**Figure 3:**
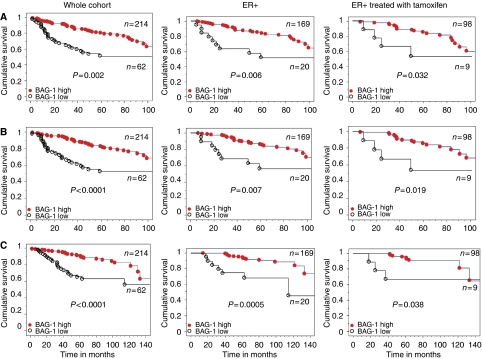
Relationship between nuclear BAG-1 protein expression by immunohistochemistry and patient outcome. Kaplan–Meier analyses (log-rank test) for (**A**) local recurrence, (**B**) distant metastases and (**C**) breast cancer-specific death in the whole cohort, ER+ subgroup and ER+ patients treated with tamoxifen stratified by high (•) and low (○) BAG-1 expression.

In the whole cohort (*n*=276), high nuclear BAG-1 expression was associated with a favourable prognosis for all measures of outcome in univariate analysis: local recurrence (*P*=0.002), distant metastases (*P*<0.0001) and breast cancer-specific death (*P*<0.0001, Table 3A and Figure 3). Furthermore, high nuclear BAG-1 expression was also an independent predictor of outcome in multivariate analysis for distant metastases (*P*=0.0455, Table 3B) but not for local recurrence or death. To assess whether BAG-1 was an independent prognostic variable and not the result of confounding by other variables, Cox proportional hazards models were constructed with step-wise removal of redundant variables until resolution. The resolved multivariate model is presented in Table 3C. High cytoplasmic expression of BAG-1 was also associated with improved outcome for local recurrence (*P*=0.0092), distant metastases (*P*=0.0013) and death (*P*=0.0046) on Kaplan–Meier univariate analysis, but was not significant in multivariate analysis.

Given the relationship between BAG-1 expression and ER status, we next assessed the association with outcome in the ER+ and ER− subgroups. Within ER-positive tumours (*n*=189), high nuclear BAG-1 expression was an independent predictor of outcome in both univariate and multivariate analyses (Figure 3 and Table 3D). In the multivariate model employed, which incorporated standard pathological indicators of outcome: tumour size, grade, nodal status, PR and HER-2, the resolved model, which eliminates redundant variables, retained HER-2, PR and BAG-1 (Table 3E). Cytoplasmic staining was not significant in univariate analysis in this group of patients. In the smaller subgroup of ER-negative tumours (*n*=85), nuclear staining of BAG-1 was not associated with any index of outcome in univariate analysis. As our data demonstrated a strong relationship between high BAG-1 expression, ER positivity and the luminal A phenotype, we assessed whether BAG-1 expression was associated with a differential response to adjuvant tamoxifen therapy. The data reported in Figure 3 demonstrate that patients treated with tamoxifen (*n*=107), whose tumours had a high nuclear BAG-1 expression, showed an improved outcome in univariate Kaplan–Meier analysis for local recurrence (*P*=0.032), distant metastases (*P*=0.019) and breast cancer-specific death (*P*=0.038).

### BAG-1 overexpression and antioestrogen sensitivity *in vitro*

**Figure 4:**
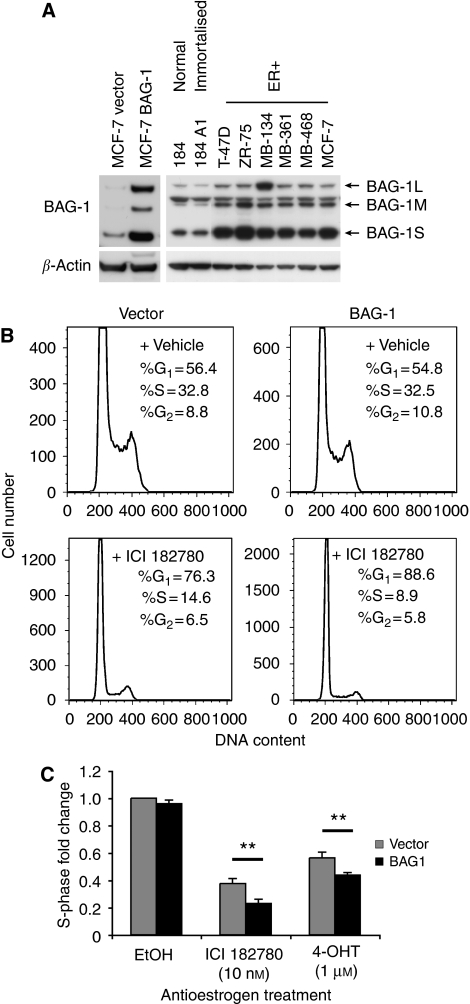
Effects of modulating BAG-1 expression on antioestrogen sensitivity in MCF-7 breast cancer cells. (**A**) Immunoblot analysis of cell lysates from MCF-7 retrovirally infected pools stably overexpressing BAG-1 wild type, two normal breast epithelial and six ER+ breast cancer cell lines. *β*-Actin was used as a loading control. Each of the three major BAG-1 protein isoforms are indicated: BAG-1L, BAG-1M and BAG-1S. (**B**) Representative DNA histograms of MCF-7 cells stably overexpressing BAG-1 compared with control cells after treatment with 10 nmol l^−1^ ICI 182780 or vehicle for 24 h. Differences in scale are due to slight differences in the number of events recorded. (**C**) Proliferating MCF-7 cells stably overexpressing BAG-1 were treated with 1 *μ*mol l^−1^ 4-hydroxytamoxifen, 10 nmol l^−1^ ICI 182780 or vehicle for 24 h. Cells were harvested and S phase was analysed by propidium iodide staining and flow cytometry. The decrease in S phase was graphed as fold change relative to vehicle-treated vector control cells. The bar histograms represent the mean±s.e.m. for replicate samples from five independent experiments. ^*^P<0.05; ^**^P<0.005.

To provide some potential mechanistic insights into the relationship between high BAG-1 expression and improved outcome in ER+ tamoxifen-treated patients, we assessed the effect of BAG-1 overexpression on oestrogen/antioestrogen sensitivity in ER+ MCF-7 breast cancer cells. A pool of high BAG-1-expressing MCF-7 cells was isolated and overexpression of the three major protein isoforms (BAG-1L, BAG-1M and BAG-1S) was confirmed by western blotting (Figure 4A). BAG-1 was also overexpressed in a panel of ER+ breast cancer cell lines compared with normal and immortalised breast epithelial cells (Figure 4A), and thus this high-expressing pool of MCF-7 cells represented an appropriate model to study the biological consequences of BAG-1 overexpression and was used for all further analyses.

MCF-7 BAG-1 cells were treated with 1 *μ*mol l^−1^ 4-hydroxytamoxifen, 10 nmol l^−1^ ICI 182780 or vehicle for 24 h and the percentage of S-phase cells determined by flow cytometry. Antioestrogen-induced cell cycle arrest was enhanced in MCF-7 BAG-1 cells compared with vector control (Figure 4B). Treatment of MCF-7 BAG-1 cells with the pure oestrogen antagonist ICI 182780 (*P*<0.005) or the active metabolite of tamoxifen, 4-hydroxytamoxifen (*P*<0.05), in replicate experiments demonstrated a significantly enhanced cell cycle arrest as measured by a decrease in S phase compared with control cells (Figure 4C).

## Discussion

The recent characterisation of molecular phenotypes of breast cancer defines biological subgroups, independent of histological type, which provides a further insight into the disease at a functional level. Luminal A cancers defined by the presence of ER and/or PR positivity and HER-2 negativity form a favourable prognostic group. However, further defining this group may provide new insights into the underlying biology of oestrogen sensitivity/resistance and provide clinically useful markers for routine clinical practice. This study demonstrates that a high BAG-1 expression identifies a good prognosis group of cancers with a luminal A phenotype, which may have enhanced therapeutic sensitivity to tamoxifen.

We first addressed the question of identifying an association between BAG-1 mRNA expression levels and patient outcome in two independent cohorts, which are broadly equivalent to our validation cohort in terms of clinicopathological characteristics. Using serially determined cut points, we identified two populations of patients with high and low BAG-1 expressions, which correlated with patient outcome. Thus, the high BAG-1 mRNA expression, found within the top 80% of patients, is associated with a favourable outcome. Correspondingly, the frequency distribution of immunohistochemically detected BAG-1 protein expression in our clinical cohort identifies two distinct subgroups of patients of similar proportions to those identified in the gene expression profiling analyses. High BAG-1 protein expression, defined as greater than 40% positive nuclear staining of any intensity, identified 78% of patients with a good prognosis. The predictive value of high BAG-1 expression was greatest in ER+ cancer in which a high nuclear expression was an independent predictor of prognosis for local recurrence, distant metastases and death. Furthermore, for breast cancer-specific death, BAG-1 expression was of superior predictive power to tumour grade, tumour size and lymph node status. This group of patients has a strong positive correlation with a luminal A phenotype and low histological grade, which suggests that BAG-1 may be a useful surrogate marker of intact ER signalling and identifies those tumours maintaining a luminal A-differentiated phenotype. Therefore, BAG-1 is a marker with potentially useful prognostic applications in ER+ disease.

Outcome studies, published earlier, of BAG-1 expression using immunohistochemistry have shown inconsistent results but with a trend towards improved prognosis with high expression levels. However, its role as a predictive biomarker has not yet been fully defined or adequately validated. The first published study (Tang *et al*, 1999) of 140 patients included both early and metastatic disease, ER status was unknown in 38% of patients and only 35% of patients were ER+. Consequently, ER and PR were excluded from multivariate analysis, which showed that an elevated nuclear BAG-1 expression was associated with shorter disease-free and overall survival, although BAG-1 was not significant in univariate analysis. These findings were not replicated in a subsequent study by the same group of investigators (Tang *et al*, 2004). The second study of 122 patients (Turner *et al*, 2001) consisted predominantly of pre-menopausal patients (mean age, 54 years), only 41% of whom had ER+ cancers, and lymph node status was unknown in 48% of cases. In addition, well-documented prognostic indicators, such as tumour size, grade, ER, PR and HER-2, were not significant in univariate analysis. In this study, elevated cytoplasmic BAG-1 expression was associated with improved prognosis in a multivariate model that included ER, BCL-2 and stage. In a more homogeneous and representative cohort of early breast cancer, Cutress *et al* (2003) described improved prognosis in univariate analysis with high nuclear, but not high cytoplasmic, BAG-1 expression in a cohort of 138 patients, 60% of whom were ER+. All patients were treated with surgery and endocrine therapy without chemotherapy. The largest and the most recent study of 517 patients (Nadler *et al*, 2008) used image analysis-based assessment of immunofluorescent staining and found that both high nuclear and cytoplasmic expression of BAG-1 was associated with improved prognosis in the whole cohort and in lymph node-positive patients only in univariate analysis, with a strong correlation with ER, PR and Bcl-2. However, again in this study, only 52% of patients were ER+, with a predominance of large tumours (59%>2 cm), and details on histological grade and treatment were not available. Two other studies were unable to identify any association with outcome in patients treated with hormonal therapy (Townsend *et al*, 2002) or in a cohort with advanced breast cancer treated with chemotherapy (Sjostrom *et al*, 2002). There are many possible explanations for these discordant findings: differences in the composition of the clinical cohorts, incomplete clinical information, different antigen retrieval methods, differing monoclonal antibodies used in the detection of BAG-1 and divergent cut points used to determine a high or low expression. This study, therefore, confirms the findings described earlier of improved prognosis with high nuclear BAG-1 expression described by Cutress *et al* (2003) and represents a detailed analysis of BAG-1 expression and its potential relationship with therapeutic responsiveness in a large cohort of uniform histological type with well-documented clinical outcome.

The finding of improved responsiveness to tamoxifen and better patient outcome associated with a high expression of BAG-1, a pro-survival antiapoptotic protein, is somewhat counter-intuitive but is mirrored by several studies identifying the overexpression of BCL-2, a major target of BAG-1, also being consistently associated with improved prognosis in low-grade ER+ tumours (Callagy *et al*, 2006) and also in patients treated with tamoxifen (Linke *et al*, 2006). Furthermore, the strong relationship between high nuclear BAG-1 expression and improved patient outcome reported by Cutress *et al* (2003) emanated from a cohort of tamoxifen-treated patients. *BAG-1*, *BCL-2* and *ER* feature among the 16 cancer-related genes of the Oncotype Dx assay (Paik *et al*, 2004), which predicts distant failure in ER+ lymph node-negative patients treated with tamoxifen. The derived recurrence-score algorithm, which is largely weighted towards proliferation-related genes, assigns a negative value to the BAG-1 mRNA expression level, in turn supporting our observation of improved prognosis with high expression level. Several other gene expression profiling studies have identified signatures predictive of outcome in ER+ disease treated with tamoxifen (Ma *et al*, 2004; Jansen *et al*, 2005; Loi *et al*, 2008). *BAG-1* did not feature among genes within these signatures, although there is often a limited overlap in signatures between studies (Miller, 2007). Interestingly, an expression profiling study (Cleator *et al*, 2006) of pre-treatment biopsies from 40 patients treated with AC chemotherapy identified *BAG-1*, *BCL-2* and *ER* among a diverse group of 178 genes overexpressed in sensitive tumours. Thus, the potential role of BAG-1 as a predictive marker of therapeutic responsiveness to both endocrine and chemotherapy requires further investigation. This is best performed within the context of randomised clinical trials and these studies are ongoing.

The *BAG-1* gene is located on chromosome 9p12 and is expressed as three protein isoforms generated through alternative initiation sites from a single mRNA (Packham *et al*, 1997). The overexpression of BAG-1 has been described in several breast cancer cell lines (Takayama *et al*, 1998; Brimmell *et al*, 1999), with the three isoforms demonstrating differing intracellular localisations: BAG-1L is predominantly nuclear, BAG-1S is predominantly cytoplasmic and BAG-1M is present in both the cellular compartments (Brimmell *et al*, 1999). This differential subcellular localisation of BAG-1 isoforms is altered under different experimental conditions, possibly representing a regulatory mechanism for protein activity: BAG-1M relocalises from the cytoplasm to the nucleus following heat shock (Zeiner *et al*, 1999) and when bound to the glucocorticoid receptor, possibly downregulating the receptor (Schneikert *et al*, 1999). Nuclear-to-cytoplasmic relocalisation of BAG-1M has also been observed during epidermal and neuronal differentiation and during breast epithelial involution, both *in vitro* and *in vivo* (Takayama *et al*, 1998; Schorr *et al*, 1999; Kermer *et al*, 2002). BAG-1 possesses a range of pro-survival properties through its ability to interact with diverse downstream target molecules, originally described by its ability to bind to and enhance the activity of the antiapoptotic protein BCL-2 mediated by its binding to the heat-shock proteins HSP70 and HSC70 (Takayama *et al*, 1995). Differential staining and subcellular localisation of the isoforms have not been investigated in the normal breast or breast cancer and are impaired by the absence of isoform-specific antibodies, all current studies in breast detecting ‘total’ BAG-1 expression. The mechanism of overexpression of BAG-1 in breast cancer is also not known although in prostate cancer it is amplified in 7.4% of hormone-refractory cancers (Maki *et al*, 2007). Our data with normal and malignant breast epithelial cell lines confirm overexpression in ER+ carcinoma cell lines compared with normal epithelial cells. The BAG-1S isoform appeared to be preferentially overexpressed, but there was evidence for elevated expression of all three isoforms in a cell line-specific manner. The relative contribution of the respective isoforms to the relationships reported here must await further studies with isoform-specific antibodies.

A key target of BAG-1 is ER*α*, which when bound by the BAG-1L isoform increases its transcriptional activity by up to five-fold in MCF-7 cells (Cutress *et al*, 2003). The ability of high nuclear BAG-1 expression to predict improved outcome in ER+ cancer and also in those treated with tamoxifen is of potential mechanistic importance as it suggests that it may have a role in responsiveness to adjuvant endocrine therapy. In our subgroup of ER+ patients treated with tamoxifen (*n*=107), high BAG-1 expression predicted improved prognosis, which may indicate sensitivity to therapy or possibly a better definition of a good prognostic luminal A group of patients. To address the question of whether BAG-1 expression could confer enhanced sensitivity to antioestrogen treatment *in vitro*, we analysed cell cycle arrest in BAG-1-overexpressing MCF-7 cells. The data presented here demonstrate a significant increase in sensitivity to the induction of cell cycle arrest by both tamoxifen and the pure steroidal antioestrogen ICI 182780.

As antioestrogen therapy, targeted at oestrogen synthesis (aromatase inhibitors), or the ER (tamoxifen), is the single most-effective treatment for women with hormone receptor-positive disease, the ability to predict likely success or failure of these therapies would enable potential alternative therapeutic strategies to be targeted to a group of patients most likely to fail on tamoxifen or aromatase therapy up-front or at an earlier stage in treatment, which may result in improved outcome. The ability of BAG-1 to predict responsiveness to antioestrogen therapy now merits further investigation by examining the relationship between expression and response in large randomised clinical trials of endocrine therapy in ER+ patients.

In summary, we have demonstrated that the high BAG-1 expression is associated with the luminal A phenotype, is an independent predictor of outcome and may indicate enhanced responsiveness to tamoxifen in ER+ invasive ductal carcinoma. These effects may be related to the ability of BAG-1 overexpression to confer increased sensitivity to antioestrogens *in vitro*. These findings suggest that BAG-1 immunohistochemistry may have a role in a routine pathology setting as a marker for better defining luminal A breast cancers and as a therapeutic response marker for ER-targeted therapy with tamoxifen or aromatase inhibitors.

## Figures and Tables

**Table 1:** Association between BAG-1 mRNA expression and breast cancer outcome

	**High BAG-1 expression *n* (%)**	**HR**	**95% CI**	***P*-value**
*(A) Cox univariate analysis for high BAG-1 expression from publicly available gene expression data sets*				
*Wound/NKI*				
(van de Vijver *et al*, 2002)	234/295 (79.3%)	0.439	0.277–0.697	0.0005
*Naderi*				
(Naderi *et al*, 2007)	108/135 (80%)	0.412	0.212–0.843	0.0151
				
*(B) Cox multivariate analysis for the Wound cohort (*n=*295)*				
Grade>2		2.266	1.361–3.774	0.0017
Size>20 mm		1.678	1.039–2.710	0.0343
ER positive		0.549	0.323–0.933	0.0267
HER-2 positive		2.319	1.267–4.244	0.0064
BAG-1 high		0.911	0.530–1.567	0.7363

CI=confidence interval; ER=oestrogen receptor; HR=hazards ratio.

**Table 2:** Clinicopathological features of the breast cancer cohort and association with BAG-1 expression

	**Nuclear BAG-1**			**Cytoplasmic BAG-1**		
	**Positive**	**Negative**	***P*-value**	**Positive**	**Negative**	***P*-value**
*(A) Clinicopathological characteristics and associations with BAG-1 expression*						
*Age*						
>50	135	39	0.979	135	39	0.252
<50	79	23		85	17	
						
*Grade*						
1 and 2	133	17	<0.0001	129	21	0.005
3	81	45		91	35	
						
*Size*						
>20 mm	81	31	0.086	82	30	0.027
<20 mm	133	31		138	26	
						
*Nodal status*						
Positive	90	30	0.424	98	22	0.429
Negative	121	32		119	34	
						
*HER-2*						
Positive	31	20	0.001	32	19	0.001
Negative	180	40		184	36	
						
*ER*						
Positive	169	20	<0.0001	166	23	<0.0001
Negative	44	41		52	33	
						
*PR*						
Positive	146	13	<0.0001	141	18	<0.0001
Negative	67	49		78	38	
						
*CK5/6*						
Positive	19	14	0.003	23	10	0.127
Negative	195	48		197	46	
						
*Triple negative*						
Positive	25	23	<0.0001	31	17	0.0039
Negative	187	37		186	38	
						
*(B) Treatment and survival data*		n(%)				
Endocrine therapy		144/292 (49.3)				
Chemotherapy		111/292 (38.0)				
Endocrine and chemotherapy		71/292 (24.3)				
Recurrences		75/292 (25.7)				
Distant metastases		68/292 (23.3)				
Deaths		67/292 (22.9)				
Breast cancer-specific deaths		52/292 (17.8)				
5-year disease-free survival		74.0%				
5-year metastasis-free survival		76.8%				
5-year breast cancer-specific survival		86.0%				

**Table 3:** Cox univariate and multivariate analyses

	***n* (%)**	**HR**	**95% CI**	***P*-value**
*(A) Whole-cohort clinicopathological variables (*n=*292), univariate analysis of breast cancer-specific death*				
Age>50	184/292 (63)	1.427	0.799–2.551	0.229
Grade>2	132/291 (45)	3.100	1.865–5.163	<0.0001
Size>20 mm	117/291 (40)	2.730	1.678–4.443	<0.0001
LN positive	125/289 (43)	3.968	2.346–6.774	<0.0001
HER-2 positive	51/273 (18)	2.459	1.463–4.134	0.0007
ER positive	192/280 (68)	0.395	0.243–0.642	0.0002
PR positive	161/282 (57)	0.238	0.140–0.406	<0.0001
BAG-1 high	214/276 (78)	0.364	0.222–0.598	<0.0001
				
*(B) Whole-cohort (*n=*276) distant metastases, Cox multivariate analysis*				
Grade>2		1.398	0.751–2.567	0.2948
Size>20 mm		1.564	0.937–2.610	0.0873
LN status		3.372	1.934–5.880	<0.0001
HER-2		1.853	1.066–3.220	0.0287
ER		0.990	0.525–1.868	0.9745
PR		0.405	0.212–0.776	0.0064
BAG-1 high		0.559	0.317–0.989	0.0455
				
*(C) Whole-cohort distant metastases, Cox multivariate analysis, resolved model*				
LN status		3.597	2.097–6.168	<0.0001
HER-2		1.973	1.158–3.361	0.0125
PR		0.329	0.186–0.584	0.0001
BAG-1 high		0.586	0.344–0.998	0.0493
				
*(D) ER*+ (n=*189) breast cancer-specific death, Cox multivariate analysis*				
Grade>2		1.529	0.600–3.896	0.3730
Size>20 mm		1.053	0.428–2.591	0.9100
LN status		1.471	0.571–3.795	0.4250
HER-2		5.578	2.036–15.286	0.0008
PR		0.293	0.119–0.721	0.0076
BAG-1 high		0.290	0.114–0.735	0.0090
				
*(E) ER*+ *breast cancer-specific death, Cox multivariate analysis, resolved model*				
HER-2		6.725	2.7–16.644	<0.0001
PR		0.239	0.104–0.547	0.0007
BAG-1 high		0.302	0.122–0.744	0.0093

CI=confidence interval; ER=oestrogen receptor; HR=hazards ratio.
